# Tuberculosis and Sexually Transmitted Infections

**DOI:** 10.3201/eid1011.030785

**Published:** 2004-11

**Authors:** Nico J.D. Nagelkerke, Sake J. de Vlas, Kelly S. MacDonald, Hans L. Rieder

**Affiliations:** * Leiden University Medical Center, Leiden, the Netherlands;; † University of Manitoba, Manitoba,Winnipeg, Canada;; ‡ University Medical Center, Rotterdam, the Netherlands;; § Mount Sinai Hospital, Toronto, Ontario, Canada;; ¶ International Union against Tuberculosis and Lung Disease, Paris, France

**Keywords:** letter, tuberculosis epidemiology, sexually transmitted infections

**To the Editor:** *Mycobacterium tuberculosis* infection is a necessary, but not sufficient, cause of tuberculosis (TB). Infection with HIV is the strongest known risk factor for disease progression to TB. In the absence of HIV infection, disease develops in 5% to 15% of infected persons. Unfortunately, the process of progression to disease is poorly understood. We hypothesize that, in addition to HIV, another sexually transmitted infection (STI) also increases such disease progression. Identification of this STI might suggest new approaches to disease control.

Several associations between the risk for TB and lifestyle factors have been identified (*1*). For example, unmarried persons are at higher risk than married persons. A correlation between TB and body mass index has also been shown; the risk of tuberculosis decreases as body mass index increases. Whether these risk factors reflect an increased risk for tuberculous infection or an increased risk for disease progression is not clear, however.

Risk factors for tuberculous disease progression itself have also been studied. Several medical conditions, e.g., hemophilia, kidney disease requiring hemodialysis, HIV infection, diabetes, some kinds of cancer, and silicosis, increase the risk of progression to disease (*1*). Another consistent finding is that age affects risk. Infants and children with immature immune systems appear to be at high risk for developing tuberculous meningitis. After the immune system matures, children appear to be rather resistant to disease progression until puberty; only 1% to 3% of infections progress to manifest disease, and pulmonary TB is rare. This resistance is largely lost after puberty, an association that seems causal. Dubos and Dubos noted "In the majority of girls, pulmonary lesions were first found in the age group of 13 to 15 years, with a striking relation to the onset of menses" (*2*). Evidence that sex hormones play a role in this loss of resistance is shown by a study on the effects of male castration on longevity (*3*). This study found that castrated, mentally handicapped patients, in the first half of the 20th century in Kansas, outlived patients who were not castrated by more than 10 years, mostly due to measurably lower TB death rates. Although this finding may reflect the direct effect of sex hormones on the immune response to tuberculous infection, we believe that it is more consistent with exposure to an as yet unidentified STI. The STI hypothesis not only explains the mechanisms behind the lifestyle risk factors discussed above, namely by confounding with sexual behavior, but also why TB has ceased to be a major cause of death in Western societies.

In the Netherlands, deaths due to TB declined consistently during the first half of the 20th century. During World War II, however, deaths due to TB almost doubled, even before living conditions deteriorated. After the war, deaths due to TB plummeted, falling almost 10-fold between 1945 and 1955, essentially before the advent of effective chemotherapy (*4*). Similar declines in deaths due to TB were observed in other industrialized countries during this period. No satisfactory explanation has been given for this pattern. Only a drop in TB progression rates could likely account for this decline because, as for much of Europe, the early postwar period was a time of scarcity and housing shortages, which rules out decreases in crowding and transmission as plausible explanations. This scarcity would also seem to exclude nutritional factors as a probable cause of falling disease progression rates. However, this epidemiologic history is very similar to that of STIs, e.g., syphilis (*5*) and may not be coincidental.

Historic data on age-specific deaths caused by TB from Massachusetts, 1880–1930 (*6*), show that deaths among women tended to peak at lower ages than deaths among men, which is similar to patterns of STI prevalence. This "young women, older men" pattern is found in most populations in which TB is endemic and appears to be caused by age- and sex-specific differences in risk of disease progression, because these differences are not found in TB infection prevalence (*7*). Such a pattern would seem more consistent with our STI hypothesis than with a direct hormonal effect on TB disease progression rates.

The association between TB and sexual behavior has rarely been studied, except within the context of HIV infection. In one study, conducted in Los Angeles, many HIV-negative TB patients reported high-risk sexual behavior (*8*), but in the absence of a control group, this finding provides only anecdotal support of our hypothesis. Recent evidence comes from a study on prison inmates in the United States in which inmates who reported a history of TB also reported higher sexual risk factors than those without such a history, although confounding by HIV infection cannot be entirely ruled out (*9*).

Which pathogen may be responsible for the other STI? The association of susceptibiblity risk with hemophilia and hemodialysis suggests that it is a filterable agent, for example, one of the many herpesviruses. Many of these are sexually transmitted, and some, e.g., Epstein-Barr virus and cytomegalovirus, have immunosuppressive properties and infect macrophages, cells that are key in the immune response to *M. tuberculosis*. Viral strategies of evading the immune system inside these cells may well create a niche for *M. tuberculosis* (*10*).

Our hypothesis could be refuted or corroborated in several ways, for example, by a case-control study of HIV-negative patients infected with tuberculosis. If this study refutes our hypothesis, the idea that sex hormones play a direct role in the immune response to *M. tuberculosis* would be supported. Such findings might also provide possibilities for drug development. However, if case-control studies support our hypothesis, attempts should be made to identify the pathogen.
